# *Satureja Khuzestanica* Mediated Synthesis of Silver Nanoparticles and Its Evaluation of Antineoplastic Activity to Combat Colorectal Cancer Cell Line

**DOI:** 10.22037/ijpr.2020.113275.14201

**Published:** 2020

**Authors:** Zahra Mohammadi-Ziveh, Seyed Ali Mirhosseini, Hamideh Mahmoodzadeh Hosseini

**Affiliations:** a *Applied Biotechnology Research Center, Tehran Medical Sciences, Islamic Azad University, Tehran, Iran. *; b *Department of Genetics, Faculty of Advanced Science and Technology, Tehran Medical Sciences, Islamic Azad University, Tehran, Iran. *; c *Applied Microbiology Research Center, Systems Biology and Poisonings Institute, Baqiyatallah University of Medical Sciences, Tehran, Iran.*

**Keywords:** Biosynthesis, Colorectal cancer, Nanoparticles, Satureja khuzistanica, Apoptosis, Silver nanoparticle

## Abstract

Several formulations of herbal plants have been extensively applied to treat diseases. *Satureja khuzistanica *(*S. khuzistanica)* is an Iranian traditional plant with a wide range of benefit effects on different diseases. In this study, we aimed to prepare silver nanoparticles from *S. khuzistanica *via the green synthesis method and investigate the anti-cancer effects on the HT29 cell line. To synthesize Ag-*S. Khuzistanica,* 50 mL* S. khuzistanica *extract and 1 mM AgNO3 were mixed and shaken at room temperature for 72 h. To determine the Ag-*S. Khuzistanica *nanoparticle characterization, XRD, FTIR, and TEM methods were done. In addition, MTT assay and real time PCR and annexin V/PI staining were performed to investigate the cytotoxicity, *bcl-2* and *bax* gene expression and percentage of apoptotic cells. Our findings showed that Ag-*S. khuzistanica *is a spherical crystalline nanoparticle with the size less than 100 nm. MTT analysis showed that 375, 750, 1500 and 3000 µg/mL *Ag-S. Khuzistanica *significantly decreased the cell viability of HT29 cells. Ag-*S. khuzistanica *significantly reduced *bcl-2* and increased apoptotic index expression at 375, 750, 1500, 3000 µg/mL *Ag-S. Khuzistanica *in a dose-dependent manner. Furthermore, cell staining with Annexin V/PI showed that treating Ag*-S. Khuzistanica *led to increasing in apoptotic cells. In conclusion, the formulation of Ag-*S. khuzistanica *has the apoptotic properties on the colorectal cancer cell line.

## Introduction

Today, cancer is known to be the leading cause of death all around the world (1). Cancer is a set of several diseases which are related but each has a different treatment. Colorectal cancer is one of the most common cancers in the world. According to the American Cancer Society’s 2019 statistics, 145,600 new cases of colorectal cancer were diagnosed in the USA that 78,500 cases were male and the rest are females. Colorectal cancer is asymptomatic in the early stages. In the late stage, patients suffer from the pain in the abdominal and rectal bleeding (2). The therapeutic strategies for colorectal cancer treatment such as surgery, radiotherapy, chemotherapy and targeted therapy have been used. Surgery is the appropriate option for treating non-metastatic cases of colorectal cancer. In the metastatic cases, chemotherapy and targeted therapy are done but they have some disadvantages including systematic toxicity and being invasive (2, 3). Therefore, to improve the cancer treatment strategy, many researchers are trying to develop new drugs and anti-cancer compounds. Herbal plants belong to the traditional drugs that have been used from the past.

The application of nanotechnology and nanobiotechnology strategy for designing and improving medical materials is common (4). Nanotechnology is the science of using nanomaterials in various fields such as medicine, electronic, chemistry, biology, phisics, pharmaceutics and etc. Nanoparticles are the materials frequently applied in the medical field for both therapy and diagnosis. In addition to the medical field, nanoparticles are being utilized in various industries in different forms such as nanowires, nanotubes, fullerene nanoparticles, and quantum dots (5). In the nanoscale, materials show the different and specific physicochemical behaviors because of Due to the higher surface-to-volume ratio. Nanomaterials have a discrepant morphology, size, charge and surface chemistry compared to native material (6). Nanoparticles have the size lower or equal to 100 nm. There are two main classes of nanoparticles called organic and non-organic nanoparticles. Chemical, physical and biological strategies are protocols to fabricate nanoparticles. Today, biological methods have become more widely used due to their simplicity, availability, affordability, and biocompatibility. In addition to ecofriendly, the toxic and hazardous substances are limited in the biological procedures (6). 

There are two main classes of nanoparticles called organic and non-organic nanoparticles. Metallic nanoparticles belong to inorganic class that are used in numerous medical and pharmaceutical area including, cellular drug delivery, gene delivery, drug delivery, controlled release of drugs, wound processing, food industry, sensor, imaging, thermal therapy and etc.(7). However, it is reported that metal nanoparticles could result in genotoxicity and cause genetic alteration such as mutations, structural chromosomal abnormalities, DNA damage. This event is the important disadvantage of metal nanoparticles (8). Gold, silver, copper, titanium, zinc and iron are the metals that are commonly utilized for medical applications (9). Silver nanoparticles are being used frequently for several applications such as pharmacology, environmental remediation, medicine, electronics, medicinal devices, biotechnology, magnetic fields, energy, and engineering, food industries, and cosmetics (5, 10). Moreover, silver nanoparticles showed potent therapeutic properties as anti-cancer, anti-parasite, bactericidal, fungicidal, antioxidant, anti- inflammatory, anti- proliferative and anti- angiogenic. The anti-bacterial and anti-tumor properties of Silver nanoparticles pave the way for their wide range usage in cosmetic and drug formulation (11). In addition; silver nanoparticles possess the high capacity to create linkage with environment and its compound. There are discrepant protocols to prepare and synthesize the silver nanoparticles such as electrochemical, physical, photochemical and sonochemical reduction, as well as heat evaporation. These procedures are difficult for using in the industrial applications because of the complicated product purification processes (12, 13). Due to low toxicity and high yield, nanoparticle synthesis based on biological compounds, such as plant extracts, bacterial and fungal materials and algal extract are broadly used (9). Green synthesis or green chemistry is an appropriate, safe, simple, eco-friendly, and efficient strategy for synthesizing nanoparticles using biological compounds such as plant extract , fungi, algae, bacteria and probiotic cell lysate (14-17). In this method, natural components can reduce the metal ions to generate metal nanoparticles (11, 18). 

*S. khuzistanica *Jamzad (Marzeh Khuzestani in Persian, family of Lamiaceae) is an endemic plant of the southern of Iran. This plant is a subshrub, branched stem about 30 cm high, densely leafy, and broadly ovaiate-orbicular covered with white hairs. It is used as a folk medicinal plant, because of its therapeutic value as an analgesic and antiseptic propertie. Active ingredients of *S. khuzistanica *essential oil (SKEO) are carvacol antioxidant and flavonoids with anti-oxidant and anti-thyroid properties(19-21). The SKEO has anti-inflammatory properties, ameliorates progression of diabetic nephropathy in uninephrectomized diabetic rats  and improves inflammatory bowel disease by reducing oxidative stress biomarkers (22-24). The extract also improves the reproductive potential of normal and cyclophosphamide treated male rats with enhancement of body antioxidant potency (25). 

Here, we proposed to use *S. khuzistanica *extract as a natural material to prepare silver nanoparticle via green chemistry strategy, and surveyed the effects of its anti-apoptotic properties on the colorectal cancer cell line.

## Experimental

*Synthesis of S.khuzistanica nanoparticles*

*S. khuzistanica *was prepared from Khorramabad in a southwestern Iran in April and confirmed by the Research Institute of Forest and Rangelands (Tehran, Iran) (Voucher number:S337IMPSIR)*. *One hundred grams of fresh leaves of* S. khuzistanica *was washed with distilled water, boiled for 30 min, and centrifuged at 5000 × g for 20 min to obtain the supernatant. *S. khuzistanica extract* was filtered using Whatman No.1 filter paper. To prepare the Ag-*S. khuzistanica , *50 mL of *S, khuzistanica *extract was mixed with 1 mM AgNO3 (Sigma-Aldrich, Germany ) in a dark bottle for 72 h and stirring was applied at 150 rpm for 24 h at room temperature. AgNO3 solution was tested in the same condition as a negative control. Following the incubation time, the solution was centrifuged twice at 3500 rpm for 20 min at room temperature. Ultimately, the pellet was washed twice by applying phosphate buffer saline (PBS) and lyophilized by freeze-dryer (Christ, Germany) for 24 h and used for further studies (26, 27).

*Characterization of Ag-S. khuzistanica*

*Transmission electron microscopy imaging*

To determine the size and morphology of Ag-*S. khuzistanica,* transmission electron microscopy (TEM) (Leo 906, Germany) was utilized at 100 kv.

*FTIR analysis*

To recognize the active biochemical and functional groups, FTIR spectroscopy (Spectrum Two, PerkinElmer, Japan) was done for *Ag-S. khuzistanica. *(28). 

*XDR*

To detect the nano-scale size of particles and polycrystals, XRD (Equinox3000, Intel, France) assay was carried out by scanning angle of 5 to 118 degrees, wavelength 1.54187 Å, voltage 40 KW, and 30 mA (28). 

*Cell culture*

The HT29 cell line was purchased from the Pasture Institute (Tehran, Iran). Cells were cultured in RPMI1640 medium containing 10% fetal bovine serum (FBS), 100 U/mL penicillin, and 100 mg/mL streptomycin. The culture condition was at 37 °C, under 5% CO_2 _atmosphere. All reagents used for cell culture were obtained from Gibco, Australia.

*Cell viability test*

To assess the cell proliferation and cytotoxic impact of *Ag-S. khuzestanica *on HT29 cells, MTT method was carried out. Briefly, 8 × 10^3^ HT29 cells were seeded to each well of a 96 well plate including 200 mL complete medium (RPMI1640 with 10% FBS and 1% antibiotic) under the culture condition. After overnight incubation, the medium of each well was exchanged with 100 μL of complete medium and the cells were exposed to various concentrations of *Ag-S. khuzestanica *including 23.43, 46.8, 93.75, 187.5, 350, 750, 1500 and 3000 µg/mL. PBS was tested as a negative control. After 24 h, the culture medium of each well was exchanged with 100 µL RPMI 1640 without FBS. Then, 20 µL of 3-(4, 5-dimethylthiazol-2-yl)-2,5diphenyltetrazolium bromide (MTT) (5 mg/mL) (Sigma-Aldrich, Germany) was added to each well. After 4 h of incubation at 37 °C and 5% Co_2 _in a dark place, the culture medium was removed and 100 µL dimethyl sulphoxide (Sigma-Aldrich, Germany) was added. ELISA reader (Biorad, USA) was applied to record the optical density of each sample at 570 nm. All concentrations were tested three times.

*Gene expression analysis*

To evaluate the effect of* Ag-S. khuzestanica *on the expression of *bax* and *bcl-2*, real-time PCR was performed. Briefly, 5 × 10^5 ^HT29 cells were treated with 375, 750, 1500 and 3000 µg/mL of *Ag-S. khuzestanica *for 24 h. The cells were trypsinized and detached. Then, the RNX-Plus kit (Cinnagen, Iran) was applied following the manufacturer’s instruction to extract the total mRNA. The nanodrop spectrophotometer (Thermos, USA) and electrophoresis on the 1.5% agarose gel were applied to evaluate each isolated mRNA in terms of quantity and quality. The related cDNA of each mRNA was synthesized using cDNA synthesis kit (Pishgam Biotech, Iran), by random hexamer, oligo dT primers, and Random Hexamer reverse transcriptase. Subsequently, the relative expression of *bax* and *bcl-2* genes was determined using real-time PCR by the specific primers (Table 1). The *B*-*actin* was tested as a housekeeping gene. 1 µL of cDNA was amplified in 20 µL mixture reaction solution containing 2Xcyber green solution and 10 pmol of described primers. Real-time PCR procedure was done by Corbett Rotor-Gene 6000 real-time PCR cycler (Qiagen Corbett, Hilden, Germany) with an initial denaturation step of 3 min at 95 °C, 40 cycles of 30 s at 95 °C, 30 s at related annealing temperature for each gene and 30 s at 72 °C. The relative expression was calculated by Rest 2009 (Qiagen, USA) (29).

*Flow cytometry analysis*

To determine the apoptotic cell percentage after *Ag-S. khuzistanica *treating of HT29 cells, flow cytometry method was performed. To perform AnnexinV/PI staining, 1 × 10^6^ cells were exposed to 375, 750, 1500 and 3000 µg/mL of *Ag-S. khuzistanica *for 24 h. After detaching the cells with Trypsin/EDTA (0.25%) and washing them with calcium-binding buffer (1 ×), Annexin V/FITC solution was added to cells. After 20 min incubation at 4 °C, cells were washed with calcium-binding buffer (1 ×) and then mixed with 10 μL PI solution and incubated at 4 °C for 10 min. Finally, the stained population was measured using a flow cytometer machine (Bio-Rad, USA).

*Statistical analysis*

Statistical analysis of findings was done by the non-parametric Mann Whitney test using SPSS software. *P*-value < 0.5 was set as significant. The statistical analysis of gene expression was carried out by Rest 2009 (Qiagen, USA).

## Results

*TEM result*

**Figure 1:**
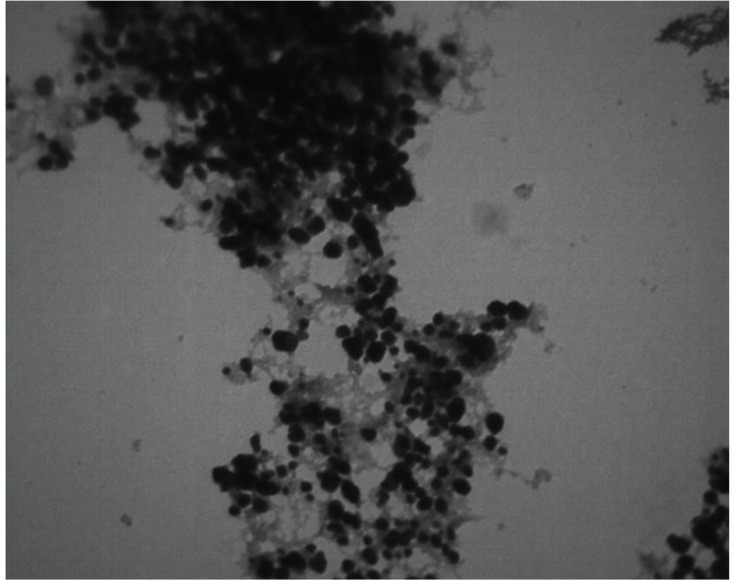
The transmission electron microscopy image of Ag-S*. khuzistanica *  at 560,000 magnification

A microscopic property of Ag*-S. khuzistanica *nanoparticles is shown in (Figure 1) with 560,000 magnifications. The size of nanoparticles was less than 100 nm. 

**Figure 2:**
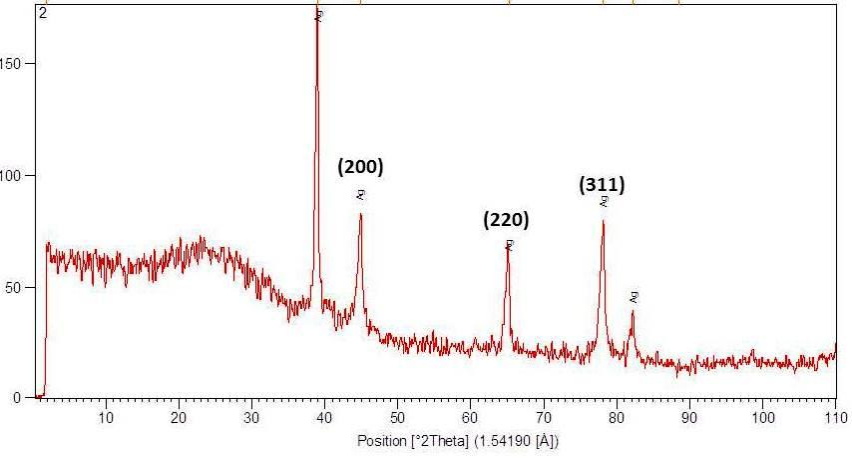
XRD pattern of Ag-*S. khuzistanica*

*XRD findings*

(Figure 2) shows the XRD pattern of *Ag-khuzistanica. *The peaks at 46.25, 67.18, and 77.52 match the 200, 220, and 311crystalline structure, in the respective order. These peaks represent the presence of Ag crystals in the *Ag-S. khuzistanica *solution. Other peaks relate to additional compounds with crystalline structure in the examined solution. Our findings showed that Ag exists in crystalline form in the center of a cube with multiple facets. 

*FTIR results*

**Figure 3:**
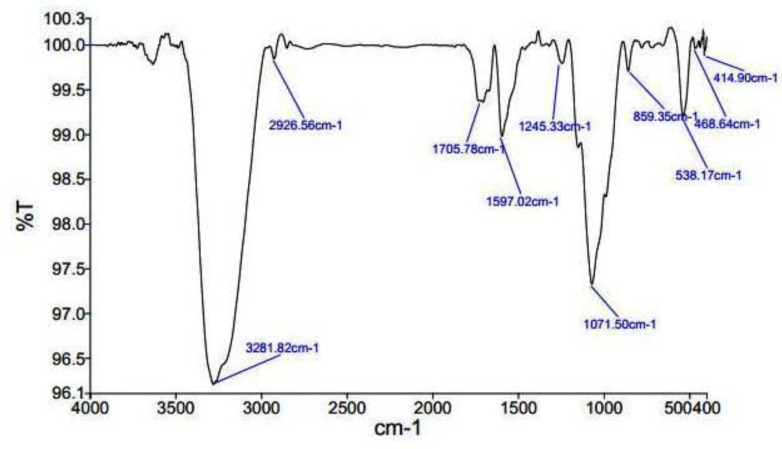
FTIR spectroscopy results of Ag-S*. khuzistanica*

As (Figure 3) shows, the existence of various peaks shows the various functional groups in various situations. In the 4^th^ area, which ranges from 1500 to 400, it is exclusive to each composition and is considered as the fingerprint of the composition. Existence of 3281/82 band is relevant to hydroxyl and N-H groups. 2926/56 band refers to the presence of C-H and methylene groups. Furthermore, 1071/50 band represents the presence of Fluoride-Aliphatic compounds and Cyclic-Ethers and cyclic compounds with C-N. 538/18 band relates to Iodide-Aliphatic compositions and 895/35 band related to Peroxide compounds and C-O-O stretch. Moreover, the presence of 1245/35 band indexes to Epoxides and oxidant compounds.1597/42 band indicates the first and second type amid functional groups. 1705/78 indicates carboxylic-Acid compounds and Ketones. 468/64 band is showed Aryl-Disolphyde. Infrared spectroscopy studies have shown that predicted biological groups have high attaching tendency to metals by covering them. These biological groups also prevent from particle oligomerization. This event causes their stability in the environment. 

*Cell viability analysis*

**Figure 4:**
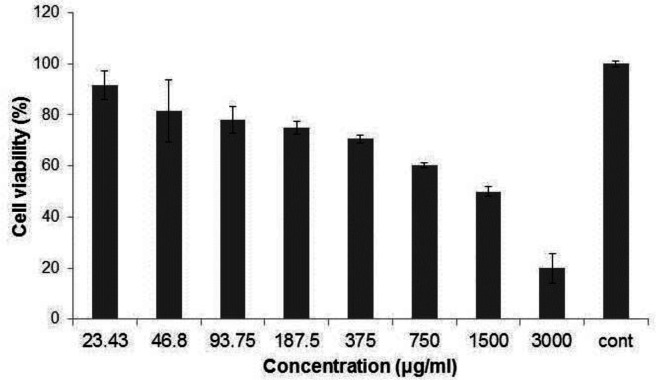
The percentage of cell viability after exposing HT-29 cell to Ag-S*. khuzistanica*

In order to identify the cytotoxic impact of *Ag-khuzistanica *on HT29 cells, MTT assay was done. As shown in (Figure 4), exposing to 23.43, 46.8, 93.75 and 187.5 µg/mL *Ag-khuzistanica *showed no statistical cytotoxic effect on the HT29 cells compared to negative control. However, 375, 750, 1500, 3000 µg/mL *Ag-khuzistanica *resulted in a decrease of cell viability percentage (70.5, 60.3, 49.9 and 19.8% viable cell, respectively) compared to a negative control (100%) (*P *< 0.05).

*Gene expression assay*

**Figure 5:**
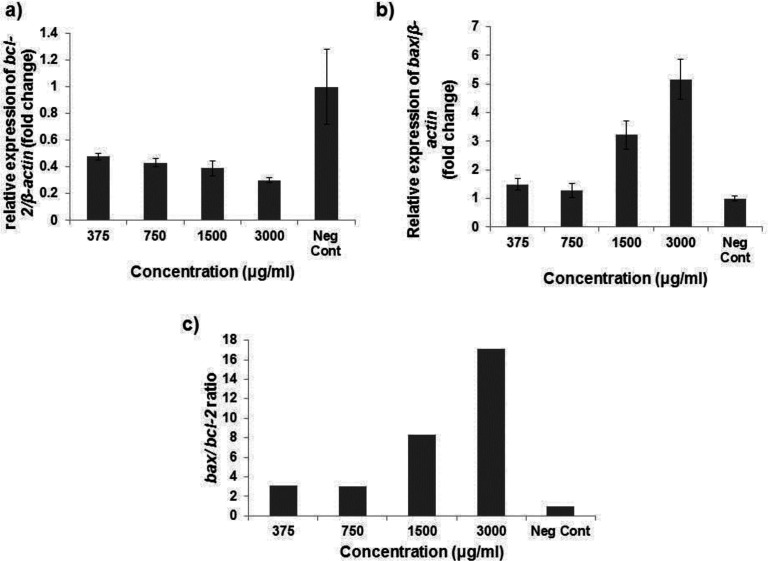
(a) Relative gene expression of *bcl-2* and *bax* (b) after exposing HT-29 cell to Ag-S*. khuzistanica *(c) the apoptotic index ratio after exposing HT-29 cell with Ag-S*. khuzistanica*

As shown in (Figure 5a) , our findings demonstrated the significant attenuation of *bcl-2* mRNA expression ratio after exposing HT29 cells to 375, 750, 1500 and 3000 µg/mL Ag*-khuzistanica *(0.48, 0.43, 0.39 and 0.3 fold, respectively) (*P* < 0.05). Moreover, the mRNA expression ratio of *bax* gene was significantly increased after treating HT29 cells with 1500 and 3000 µg/mL Ag*-khuzistanica *(3.2 and 5.1 fold, respectively) (*p* < 0.05) (Figure 5b). In addition, we observed that apoptotic index, *bax/bcl-2* expression ratio, was considerably increased after 375, 750, 1500 and 3000 µg/mL Ag*-khuzistanica *(*p* < 0.05) (Figure 5c). 

*Flow cytometery analysis*

**Figure 6:**
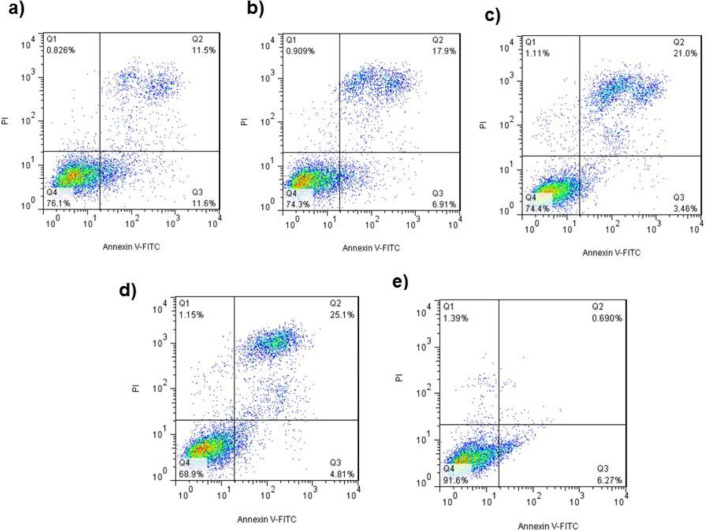
(a) Annexin/PI staining of exposed HT-29 cells to Ag-S*. khuzistanica *at 375 µg/mL, (b) 750µg/mL, (c) (d)1500 µg/mL, 3000 µg/mL and (e) Negative control

As shown in Figure 6, treating with 375, 750, 1500 and 3000 µg/mL Ag*-khuzistanica *increased the apoptotic cells, significantly in comparison with control group. Viable cells are colorless (Q4-distinct), early apoptotic cells indicated green fluorescent, which was relevant to Annexin V (Q3-distinct), and late Apoptosis indicated both colors of Annexin-V and PI (Q2) and Necrotic cells take PI (Q 1). 

## Discussion

In this study, AgNO3 was used to synthesize silver nanoparticles from *S. khuzistanica*. During the synthesizing period, the color of mixture containing silver nitrate and *S. khuzistanica *extract changed. Previous studies reported that creating a new color is due to the production of Ag-nanoparticles that leads to stabilizing and restoring the silver nitrate and *S. khuzistanica *agents*. *After adding Ag ^+^ ions to *S. khuzistanica  *extract, substances of *S. khuzistanica  *extract reacts with Ag^+^ and produces [Ag (*S. khuzistanica  *)] (28, 30-31). Silver is the plasma metal that has positive ions stabilized in the position and can conduct free electron. The free electrons are excited after exposing to electromagnetic wave and overall, they form plasmons. Surface plasmon resonance is due to interaction between plasmons and light visible under specific situation and is a main factor to generate optical spectra for viewing silver nanoparticle (15). In current study, Ag-*S. khuzistanica *compound was also assessed by the absorption at UV-visible range by a spectrophotometer. This test is significant for identifying the formation, shape, and stability of Ag-*S. khuzistanica* as a silver nanoparticle.

Herein, the FTIR spectrum was performed to investigate biochemical and functional groups involved in restoring and reducing silver ions to form silver-nanoparticles. The presence of first and second type amid groups, epoxide, and other predicted functional groups by FTIR was strong attaching tendency to metals and by covering them and prevented a particle oligomerization and cause to their stability in environment. The peaks at 46.25, 67.18, and 77.52 in XRD results correspond to the 200, 220, and 311crystalline structures, respectively. These peaks denote the presence of Ag crystals in the *Ag-S. khuzistanica *solution. Other peaks relate to additional compounds with crystalline structure in the examined solution. Our findings show that Ag exists in crystalline form in the center of a cube with multiple facets. Findings from TEM image revealed the agglomerated particles with the spherical shape. Nanoparticles tend to be aggregated that leads to their increased size. The size of *Ag-S. khuzistanica *was less than 100 nm. Our observations about morphology and the size of *Ag-S. khuzistanica *are similar to most previous studies that investigated the biofabricated silver nanoparticles on colorectal cancer cell lines (2).

Toxicity assessment is one of the main factors for cellular responses to nanoparticles (32). In addition, it is reported that silver nanoparticles create reactive oxygen species in the cancer cells leading cell damage and cell death (33). Raghunandan and co-workers fabricated gold nanoparticles and silver nanoparticles using guava leaf and clove bud extract and assessed their cytotoxicity activity against several cell lines such as HEK-293, K-562 and HeLa cell line. They observed that both types of gold nanoparticles are able to inhibit proliferation of cancer cell lines less than 10 µg/mL. They believed that the irregular morphology of gold nanoparticles is one of the important factors for its growth inhibitory effect. This shape causes to adsorb the gold nanoparticles to the extracellular surface of cancer cells and damage and be permeable them. In consistent with our study, silver nanoparticles synthesized by guava leaf and clove bud extract had no inhibitory effect on all cell lines studied that This is probably due to the spherical shape of these nanoparticles (34). 

In another study, Schneider *et al*., assessed the anti-cancer activity of five different metal nanoparticles synthesized by silver, gold, titanium dioxide, zinc oxide, copper oxide on the HT29 colorectal cancer cell line. The tested concentration of nanoparticles was 2-10 µg/mL that is lower than our concentrations. They reported that each HT29 cell adsorbed 0.02-1.39 pg/cell based on the type of nanoparticles. Finding from trypan blue staining and MTT assay showed the remarkable reduction of cell viability after treating with metal dioxide. Moreover, silver nanoparticles also induced significant decrease of cell proliferation. This effect was dose-dependent for all nanoparticles. Higher concentrations of nanoparticles led to further reduction of cell viability (35). In our study, the Ag-*S. khuzistanica *also can significantly decrease the percentage of cell viability but with a much higher concentrations. 

In addition, Schneider *et al *investigated the impacts of different nanoparticles on the cell apoptosis. They observed that silver nanoparticles, titanium dioxide nanoparticles and zinc oxide nanoparticle remarkably enhanced the percentage of cell apoptosis that was dose-dependent (35). Moreover, various previous studies reported the induction of apoptosis in the cell lines including 549 cells as a human lung carcinoma cells, HCT116 cells as a human colon carcinoma cells, and HepG2 cells as a human hepato carcinoma cells after exposing to silver nanoparticles (36-38). 

Delay and prevention of apoptotic pathways in cancer cells are common via changes in the expression of anti-apoptotic and pro-apoptotic genes causing reduced apoptotic index (*bax/bcl-2 ratio*) (39). It is believed that herbal plants induce chemopreventive properties in the cancer cells (40). *S. khuzistanica *as the medicinal plant has anti-inflammatory, anti-bacterial, anti-oxidant, and anti-cancer impacts based on its components and biological molecules (41). In the study conducted by Esmaeili-Mahani *et al*., the anti-cancer effects of *S. khuzistanica *extract on the MCF-7 cell line were reported. They observed that 150 and 200 μg/mL of *S. khuzistanica *can decrease the percentage of cell viability and increase the apoptotic index* (bax/bcl-2 *ratio) along with the activation of caspase-3 (42)*.* In addition, Yousefzadi *et al*., reported the anticancer and anti-bacterial effect of *S. khuzistanica *essential oil. They observed *S. khuzistanica *essential oil could reduce percentage of cell viability in the Vero, SW480, MCF7, and JET 3 cells with the IC50 of 31.2, 62.5, 125, and 125 mg/mL, respectively (43). In another study conducted by Satapathy and co-worker, silver nanoparticles were synthesized by plant extract (Periwinkle) named PD-AgNPs, and AgNO3 to reduce colon cancer cell toxicity of chemical materials. They observed the anti-cancer activity of PD-AgNPs at nM concentration that is less than AgNO3 and plant extract. In addition, their results showed that PD-AgNPs stimulate apoptosis via activation of p53, *bax/bcl-x* index, cytochrome c release and cleavage of PARP. Activation of caspase-3 , 9 and 8 was shown after treating HCT-116 with silver nanoparticles (44). Furthermore, they reported the increase of NK, p-JNK and c-JUN independent to quantity of p38, p-p38 and ERK (45). In addition to mitochondria dependent apoptotic properties, silver nanoparticles can induce DNA damage via releasing inflammatory cytokines including TNF-α, IL-1 and IL-6 (46). However, the mechanism and the level of silver nanoparticle effects are dependent to the chemical content of plant extract using for bio synthesis and physicochemical properties of silver nanoparticles.

To the best of our knowledge, our study is the first one to synthesize the silver nanoparticle from *S. khuzistanica extract* via green synthesis protocol. In this study, we observed that Ag-*S. khuzistanica *could induce cytotoxic impact on the colorectal cancer cells in a dose-dependent manner. In addition, this formulation can alter the mRNA expression of main genes involved in mitochondrial apoptosis. Ag-*S. khuzistanica *reduced *bcl-2* expression and increased the *bax* expression and apoptotic index in a dose-dependent manner. Furthermore, cell staining with Annexin V/PI showed that treating Ag*-khuzistanica *led to increased apoptotic cells.

**Table 1:** Sequences of Primer

**Gene**	**Forward (5’-3’)**	**Reverse (5’-3’)**	**Length (bp)**
***bax***	TGGAGCTGCAGAGGATGATTG	GAAGTTGCCGTCAGAAAACATG	95
***bcl-2***	CTGCACCTGACGCCCTTCACC	CACATGACCCCACCGAACTCAAAGA	189
***Β-actin***	TCATGAAGATCCTCACCGAG	TTGCCAATGGTGATGACCTG	180

## Conclusion

In this study, we fabricated the silver nanoparticles based on AgNO_3_ and the extract derived from the leave of *S. khuzistanica *using green synthesis method and investigated its anti-cancer activity. Our findings showed that Ag-*S. khuzistanica *is the spherical nanoparticles with the crystalline structure and its size is under 100 nm. In addition*, *infrared spectroscopy studies have been showed that predicted biological groups have been high attaching tendency to metals and by covering them and also prevented a particle oligomerization and cause to their stability in the environment. Ag-*S. khuzistanica *exerts the apoptotic effect on the colorectal cancer cell line. It can increase the apoptotic index and apoptotic cells in a dose-dependent manner. Overall, the formulation of silver nanoparticles from* S. khuzistanica *has anti-cancer activity. 
